# Successful Ablation of Epicardial Premature Ventricular Complexes Near the Great Cardiac Vein from the Left Ventricular Endocardium Despite Predictors of Failure

**DOI:** 10.19102/icrm.2017.081001

**Published:** 2017-10-15

**Authors:** Darpan S. Kumar, Peter M. Jessel, Merritt H. Raitt, Ignatius Gerardo E. Zarraga

**Affiliations:** ^1^Oregon Health & Science University, Portland, OR; ^2^Veterans Health Administration, Portland, OR

**Keywords:** Catheter ablation, great cardiac vein, mapping, premature ventricular complex

## Abstract

Mapping and ablating premature ventricular complexes (PVCs) that originate near the great cardiac vein (GCV) and anterior interventricular vein (AIV) can pose several challenges related to the advancement and positioning of catheters within these veins, the delivery of effective lesions, and the risk of collateral injury to the left coronary arteries and left phrenic nerve. When ablation of these PVCs from inside the GCV/AIV is not possible, a systematic assessment of nearby vantage points, such as the left coronary cusp (LCC) and left ventricular (LV) endocardial breakout site, should be considered, in addition to the performance of a more invasive epicardial ablation procedure via a percutaneous pericardial puncture or thoracotomy. Several electrocardiographic, anatomic, and electrogram timing features have been shown to predict the likelihood of successful ablation from a non-epicardial site, such as the LCC or LV endocardium, but none of these spots is considered to be a perfect location. The case described here in this report is a demonstration of a safe and successful ablation of GCV PVCs from the LV endocardial breakout site using adequate power and lesion duration, even when the site was 17 mm away from the putative origin, and some previously described electrocardiographic and electrogram-based predictors of success suggested the outcome would not be positive.

## Introduction

In recent years, there have been considerable advances in our understanding of the various sites from which frequent monomorphic premature ventricular complexes (PVCs) can arise, and in the different ablative strategies to eliminate such PVCs. This case illustrates some of the obstacles in mapping and ablating frequent PVCs that originate near the great cardiac vein (CGV) and reviews some strategies to overcome them.

## Case presentation

**Figure 1:**
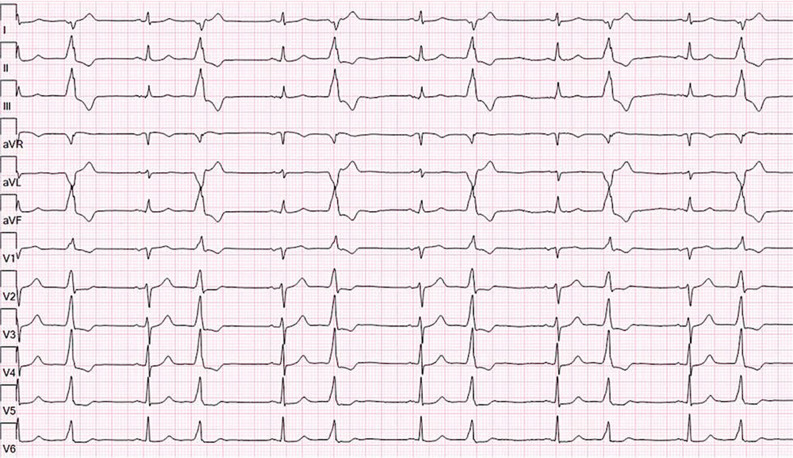
Electrocardiogram showing the patient’s frequent PVCs.

**Figure 2:**
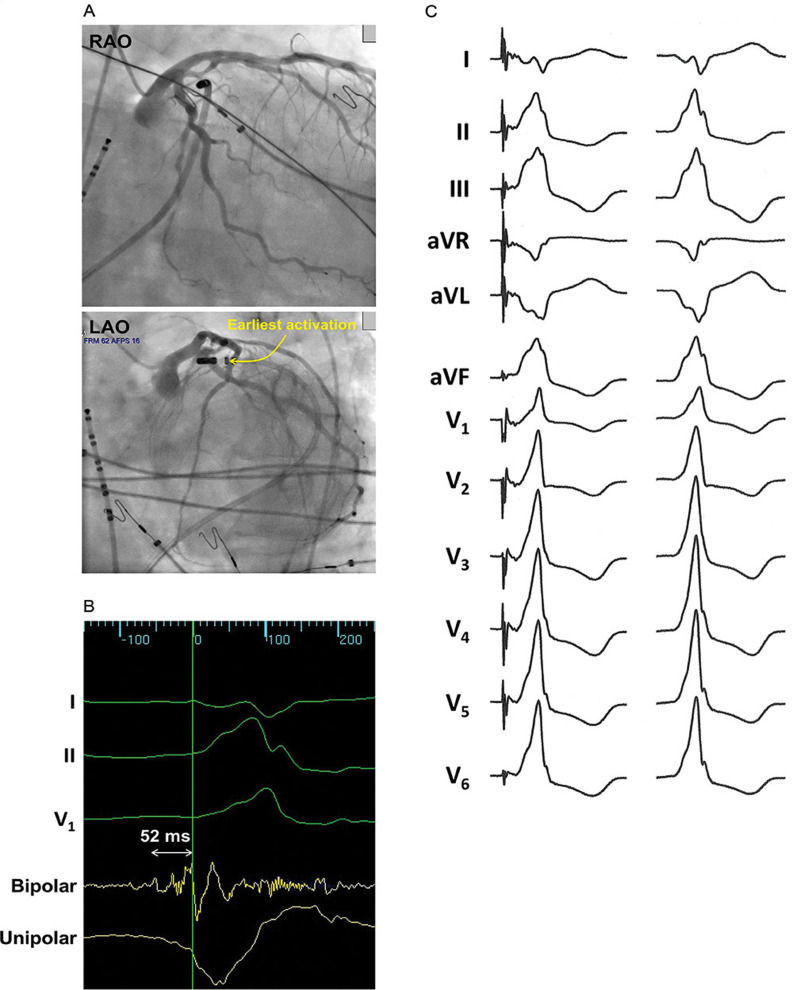
Mapping within the coronary venous system. **A:** Fluoroscopic views of the catheter inside the GCV during left coronary angiography. **B:** Local electrograms in the site of earliest activation inside the GCV. **C:** Twelve-lead electrocardiograms showing a good match between pace-mapping in the site of interest in the GCV (left) and the PVC (right).

**Figure 3:**
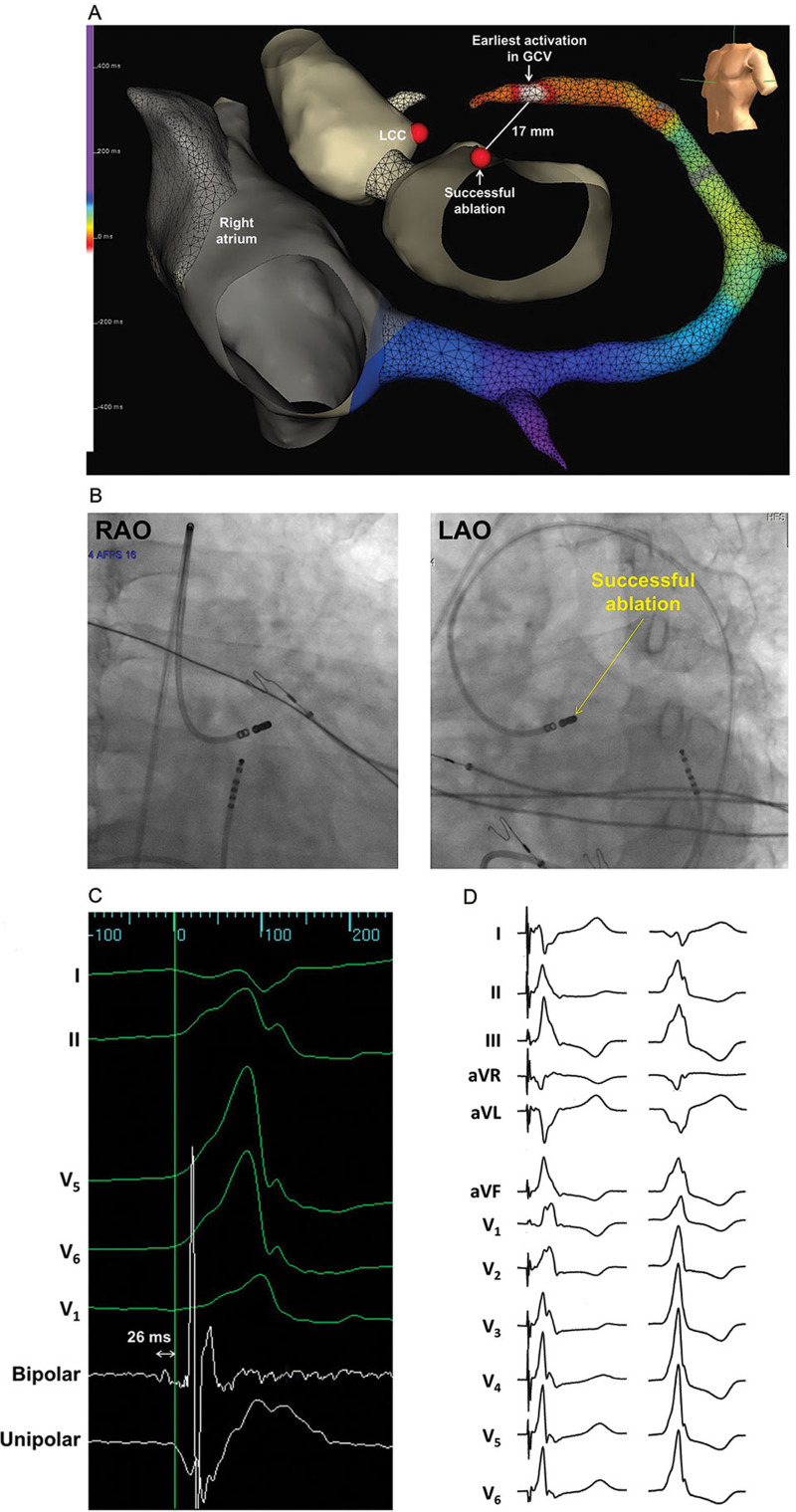
Mapping in the LV endocardium near the aorto-mitral continuity. **A:** A three-dimensional electroanatomic map showing the site of earliest activation in the GCV, the LCC, and the site of successful ablation in the LV endocardium. Courtesy of Brock I. Gambill of Abbott Laboratories (Chicago, IL, USA). **B:** Fluoroscopic views of the ablation catheter with its tip in the site of successful ablation. **C:** Local electrograms in the site of successful ablation. **D:** Twelve-lead electrocardiograms showing a poor match between pace-mapping in the site of successful ablation (left) and the PVC (right).

A 56-year-old male with hypertension, non-obstructive coronary artery disease, and non-ischemic cardiomyopathy (left ventricular ejection fraction (LVEF) of 45% to 50%) presented with symptoms of gradually progressive fatigue and intermittent lightheadedness for several months, and was found to have frequent monomorphic PVCs. A 24-hour Holter monitor revealed a PVC burden of 16%. In an attempt to suppress the PVCs, improve his cardiomyopathy, and ease his symptoms, metoprolol was started, but it had no significant effect on the PVCs. A decision was then made to pursue catheter ablation of the PVCs, with the presumption that they were a major contributor to the patient’s cardiomyopathy and symptoms. The PVC morphology (right bundle branch block (BBB) pattern and right inferior axis) pointed to an origin close to the left ventricular outflow tract (LVOT), while the maximum deflection index (MDI) of the PVC (0.61) and the presence of a Q-wave in lead I suggested an epicardial origin **(Figure 1)**.^1,2^ These features made the GCV and anterior interventricular vein (AIV; sometimes referred to as the “distal” GCV) distinctly possible sites of origin. Because of this, and the fact that the coronary venous system was readily accessible, the said venous system was mapped prior to any attempt being made to map other sites that would require percutaneous epicardial access, femoral arterial access, or transseptal puncture. Using an irrigated 3.5-mm-tip ablation catheter, a site of early activation during the PVC was found inside the GCV, near its junction with the AIV **(Figure 2A)**. Here, a low-amplitude signal was reproducibly observed to precede the PVC by about 52 ms, and pace-mapping produced a good match with the PVC **(Figures 2B and 2C)**. However, coronary angiography showed that this site was within 5 mm of the left coronary arteries, so no ablation was performed **(Figure 2A)**. Following that, a systematic search for nearby vantage points was conducted. The left coronary cusp (LCC) was mapped, as the PVC had electrocardiographic features that had been shown to predict successful ablation from the LCC—specifically, a Q-wave ratio in aVL/aVR of 1.33 and an R-wave ratio in III/II of 1.06.^3^ However, the earliest activation in the LCC preceded the PVC by 5 ms only, and was 21 mm away from the site of interest in the GCV, as measured from the electroanatomic map. Ablation in the LCC had no effect on the PVCs. The next vantage point that was sought was the left ventricular (LV) endocardial breakout site. This was found in the superior mitral annulus, close to the aorto-mitral continuity **(Figures 3A and 3B)**. The local signal here had a consistent low-amplitude electrogram that preceded the PVC by 26 ms only, and pace-mapping produced a poor match **(Figures 3C and 3D)**. However, it was 17 mm away from the putative origin in the GCV (ie, closer than the LCC), and almost directly faced this GCV site. With a power of 50 watts (W) to help ensure transmurality of the lesion, ablation was performed at the LV endocardial breakout site. No effect was seen until 25 s into the ablation, when the PVCs disappeared and never returned for the remainder of the procedure. A full minute of radiofrequency (RF) energy was delivered, followed by three full-minute consolidation lesions adjacent to the first lesion. There were no acute periprocedural complications. Six months after the ablation, the patient’s PVC burden remained very low (<1% by 24-hour Holter monitoring), and his original symptoms of fatigue and intermittent lightheadedness had resolved completely. No long-term complications related to the procedure were identified, and radionuclide ventriculography showed a normalized LVEF of 56%.

## Discussion

Cardiomyopathy resulting from frequent ventricular ectopy is now a well-accepted occurrence, and this case is yet another illustration of how successful ablation of PVCs in this situation can improve LV systolic function. Certain sites appear to have a predilection for causing frequent PVCs (eg, right and left ventricular outflow tracts, atrioventricular annuli, regions adjacent to the coronary venous system, LV summit, cardiac crux, and papillary muscles); and several electrocardiographic criteria have now been developed to predict where they can be successfully ablated, enabling operators to plan a suitable mapping and ablation strategy and counsel the patient appropriately prior to the procedure. Among these criteria are the BBB pattern, axis, precordial transition, and MDI of the PVC. For example, an MDI ≥ 0.55, a Q-wave in lead I for a PVC from the anterior LV, and a broad initial R-wave in lead VI for a GCV/AIV PVC would all be suggestive of an epicardial origin.^1,2,4^ For GCV/AIV PVCs, a right BBB pattern would point to an origin in the GCV along the basal lateral LV, from which the PVC wavefront spreads towards lead VI, while a left BBB pattern would suggest an origin in the AIV along the basal anterior LV, from which the PVC wavefront moves away from lead VI.^4^ In the case presented here, the electrocardiographic characteristics of the PVC localized it to the epicardium near the LVOT. Indeed, among all the sites that were mapped in this patient, it was a site inside the GCV that had the earliest activation and that produced the best pace-map match.

Mapping and ablating within the coronary venous system can pose several challenges. The usual diagnostic catheters can be difficult to advance to the GCV or AIV and, occasionally, a prominent valve of Vieussens adds to this difficulty. One sometimes has to resort to a much smaller catheter (eg, a 2.5-French multipolar Pathfinder^®^ catheter; Cardima, Inc., Fremont, CA, USA) to extensively map the GCV and AIV. The GCV and AIV are also in close proximity to the left coronary artery system, so even when an ablation catheter can be successfully positioned in the appropriate site in the CGV or AIV, it may not be safe to perform RF ablation. Coronary angiography must always be performed, because the risk of coronary artery injury increases substantially when the distance between the site of RF ablation and the artery is ≤ 4 to 5 mm.^5,6^ One study specifically assessed the risk of coronary artery injury from RF ablation within the coronary venous system, using 5 W to 15 W with a non-irrigated catheter, and 15 W to 25 W with an irrigated catheter, and found a 50% and 7% risk when ablation was performed ≤ 2 mm and 3 mm to 5 mm, respectively, from an artery.^6^ In the same study, cryoablation was not associated with coronary artery injury. However, the increased stiffness of cryoablation catheters, in comparison with most RF catheters, can make their advancement into the GCV and AIV a problem. Adequate power delivery is another challenge with RF ablation from within the GCV and AIV. Because of low flow and poor convective cooling within these veins, the temperature can rise rapidly and substantially during ablation, and limit the delivered power and consequently, the lesion size. This problem is particularly relevant for a PVC origin that is thought to be epicardial, but is actually deep in the myocardium, and can be circumvented by the use of an irrigated-tip catheter. Finally, the left phrenic nerve can, along its course, come close to the CGV, so capture of the phrenic nerve by high-output pacing inside the GCV can likewise prohibit safe ablation in that site.^7^ For all of the above reasons, it is not surprising that the reported rate of acute success with ablation of GCV/AIV PVCs is 43% to 70% only.^4,8,9^ For the same reasons, it is important to have alternative strategies for ablating such PVCs. These include conducting a systematic assessment of nearby vantage points, such as the LCC and the LV endocardium, with the recognition that sometimes ablation in two or more adjacent vantage points is required to eliminate the PVCs, as well as for performing a more invasive epicardial ablation via a percutaneous pericardial puncture or thoracotomy.

The proximity of a vantage point to the origin of the PVC helps to determine the likelihood of successful ablation from that vantage point. When approaching a GCV/AIV PVC from the LCC, it is helpful to understand that the part of the GCV that is closest to the LCC is to the right of the more proximal segment of the GCV. Therefore, an origin close to the LCC, as compared with one farther from the LCC, would be expected to produce a deeper Q-wave in lead aVR than in lead aVL, and a taller R-wave in lead II than in lead III. Indeed, one study showed that a distance < 13.5 mm between the earliest GCV/AIV site and the closest point of the LCC, a Q-wave ratio in aVL/VR < 1.45, and an R-wave ratio in III/II < 1.13 were all predictors, albeit imperfect ones, of successful ablation from the LCC or adjacent endocardium below the LCC.^3^ In the case presented here, the Q- and R-wave ratios of the PVC suggested an origin close to the rightmost part of the GCV, but the actual distance between the PVC origin and the LCC (21 mm) suggested that the two sites were not sufficiently close, and indeed, ablation in the LCC did not eliminate the PVC.

The LV endocardium below the aorto-mitral continuity is another vantage point for ablating GCV PVCs. In one study, all successful cases were characterized by an initial R-wave in the PVC in lead I, and an interval ≤ 7 ms between the earliest activations in the GCV and in the LV endocardium (ie, the “GCV-non-GCV interval”).^8^ When the latter was > 7 ms, ablation in the LV endocardium was unsuccessful. As a matter of fact, if the PVC origin were intramural, then a GCV–non-GCV interval ≤ 7 ms, as compared with an interval > 7 ms, would suggest that the origin was closer to the endocardium, and would explain the higher likelihood of successful ablation from the endocardium. The case presented here was unique because endocardial ablation was successful, even though the PVC did not have an initial R-wave in lead I, but rather a Q-wave, a marker of epicardial origin for PVCs arising from the anterior LV.^2^ In addition, the GCV-non-GCV interval was 26 ms. That being said, as compared with the LCC, the LV endocardial breakout site was closer to the putative origin in the GCV, and of the various sites from which ablation could be attempted, this endocardial site proved to have the lowest risk of collateral injury. The latter allowed for the use of high power, which the operators recognized was necessary to create a lesion that was sufficiently large and transmural to reach the PVC origin. A longer ablation time was likewise expected before an effect could be observed because the vantage point was some distance away (17 mm) from the putative origin. A similar case was recently described in which a microreentrant ventricular tachycardia in the basal anterior epicardial LV was successfully eliminated using prolonged ablation with 50 W in the endocardium, directly opposite to the epicardial circuit.^10^ The success of the ablation presented in this case, and the absence of associated acute or long-term complications, demonstrated the value of vantage points that are a little farther from the origin than those previously described, especially if sufficiently high power can be used safely and if ablation is allowed to continue for a longer duration than usual before it is terminated due to the absence of an observed effect.

## Conclusions

Frequent PVCs that originate near the GCV can be ablated successfully and safely from the LV endocardial breakout site, near the aorto-mitral continuity, even when the endocardial site is as far as 17 mm away from the putative epicardial origin, and some predictors of success based on electrocardiographic features and electrogram timing suggest otherwise.
